# Blasting without Drilling

**Published:** 1881-10

**Authors:** 


### BLASTING WITHOUT DRILLING.

Experiments have been recently carried out by Major Lauer, of the Austrian Engineers, at Krems, on the Danube, to show the value of his new method of blasting rocks under water. The chief feature of Lauer's system is to employ a hollow cylinder, like a gas pipe, and to place the dynamite cartridge, not as hitherto in a hole bored into the rock to be blasted, but in the cylinder in question. The cartridge only touches the surface of the rock which it is desired to shatter. The explosion of the dynamite is effected by means of electricity, and the effect is said to be greater than with the usual cartridge in a hole bored in the rock. The rock is shattered into fragments so small that a fair stream is able to wash them away without help, whereas in the case of gunpowder the rock is only split up into blocks more or less large and troublesome to remove. The Lauer system is calculated to effect a saving of fully 40 per cent. as compared with the old system.—*Scientific American.*

